# Using path analysis to test theory of change: a quantitative process evaluation of the MapSan trial

**DOI:** 10.1186/s12889-021-11364-w

**Published:** 2021-07-16

**Authors:** Sarah Bick, Helen Buxton, Rachel P. Chase, Ian Ross, Zaida Adriano, Drew Capone, Jackie Knee, Joe Brown, Rassul Nalá, Oliver Cumming, Robert Dreibelbis

**Affiliations:** 1 Department of Disease Control, London School of Hygiene and Tropical Medicine, London, UK; 2 Wexner Medical Center, Ohio State University, Columbus, OH USA; 3 WE Consult, Maputo, Mozambique; 4 School of Civil and Environmental Engineering, Georgia Institute of Technology, Atlanta, GA USA; 5 Department of Environmental Sciences and Engineering, Gillings School of Global Public Health, University of North Carolina at Chapel Hill, Chapel Hill, NC USA; 6 Ministério da Saúde, Instituto Nacional de Saúde Maputo, Maputo, Mozambique

**Keywords:** Theory of change, Path analysis, Process evaluation, Sanitation, Structural equation modelling, Complex intervention, Mozambique

## Abstract

**Background:**

Although theory-driven evaluations should have empirical components, few evaluations of public health interventions quantitatively test the causal model made explicit in the theory of change (ToC). In the context of a shared sanitation trial (MapSan) in Maputo, Mozambique, we report findings of a quantitative process evaluation assessing intervention implementation, participant response and impacts on hypothesised intermediary outcomes on the pathway to trial health outcomes. We examine the utility of path analysis in testing intervention theory using process indicators from the intervention’s ToC.

**Methods:**

Process data were collected through a cross-sectional survey of intervention and control compounds of the MapSan trial > 24-months post-intervention, sampling adult residents and compound leaders. Indicators of implementation fidelity (dose received, reach) and participant response (participant behaviours, intermediary outcomes) were compared between trial arms. The intervention’s ToC (formalised post-intervention) was converted to an initial structural model with multiple alternative pathways. Path analysis was conducted through linear structural equation modelling (SEM) and generalised SEM (probit model), using a model trimming process and grouped analysis to identify parsimonious models that explained variation in outcomes, incorporating demographics of respondents and compounds.

**Results:**

Among study compounds, the MapSan intervention was implemented with high fidelity, with a strong participant response in intervention compounds: improvements were made to intermediary outcomes related to sanitation ‘quality’ – latrine cleanliness, maintenance and privacy – but not to handwashing (presence of soap / soap residue). These outcomes varied by intervention type: single-cabin latrines or multiple-cabin blocks (designed for > 20 users). Path analysis suggested that changes in intermediary outcomes were likely driven by direct effects of intervention facilities, with little contribution from hygiene promotion activities nor core elements expected to mediate change: a compound sanitation committee and maintenance fund. A distinct structural model for two compound size subgroups (≤ 20 members vs. > 20 members) explained differences by intervention type, and other contextual factors influenced specific model parameters.

**Conclusions:**

While process evaluation found that the MapSan intervention achieved sufficient fidelity and participant response, the path analysis approach applied to test the ToC added to understanding of possible ‘mechanisms of change’, and has value in disentangling complex intervention pathways.

**Trial registration:**

MapSan trial registration: NCT02362932 Feb-13-2015.

**Supplementary Information:**

The online version contains supplementary material available at 10.1186/s12889-021-11364-w.

## Background

Process evaluations of complex interventions elucidate how and why an intervention has (or fails to have) a particular effect and which intervention components have the greatest impact on outcomes [1]. Recent Medical Research Council (MRC) guidance on evaluating complex interventions [2] recommends theory-driven approaches to evaluation that articulate the hypotheses and rationale behind interventions so they can be evaluated [3]. Public health interventions are either implicit or explicit embodiments of theory, involving the expectation that intervening will alleviate a problem via a set of intermediary outcomes and relying on a set of assumptions about how participants respond to programme activities to produce outcomes [4]. These ‘mechanisms of change’ are highly sensitive to context [4]; and recent approaches to evaluation – such as the ‘realist’ approach [5] – highlight the need for evaluation approaches that assess how intervention effects differ by contextual factors such as social group or location to understand ‘what works, for whom and in what circumstances*’* [6].

The theory of change (ToC) approach [7] depicts the hypothesised mechanisms of an intervention as a causal diagram of multiple pathways leading from activities to outcomes [8]. Each outcome in a ToC is measured by an indicator, and therefore the theory lends itself to testing using a range of analytic methods [9]. Statistical analysis of relationships between indicators in a ToC could identify mediating factors that intervene in the relationship between two components [10]. Quantitatively testing an hypothesized causal model in this way could improve our understanding of context-mechanism-outcome configurations [11], and attribute deviations from expected outcomes to theory and/or intervention failure [12]. Although ToC is recommended by official bodies concerned with clinical and complex intervention trials [2, 13], a systematic review of the uses of ToC in public health intervention evaluations found that very few have used the approach alongside trials of health impact, explored the influence of context on outcomes, or quantitatively tested the causal model [9].

Path analysis – an early variant of structural equation modelling (SEM) – is an analytical tool used to estimate relationships that account for variation among a set of observed variables, and has been suggested for use within programme evaluations [14]. Path analysis is predicated on defining a strong theoretical model that posits hypothesised linear relations between variables and reduces to the solution of one or more multiple regression analyses [15]. Path models involve two sets of variables: *exogenous* variables, whose variation is explained by factors not in the model; and *endogenous* variables, whose variation is explained at least in part by other variables in the model. Correlations among variables can be decomposed into direct and indirect (mediated) effects [16], detailing patterns of different effects in one model. A theory can then be identified as tenable among alternative theoretical models based on the ability to explain the pattern of observed relationships.

Aspects of both ToC and path analysis suggest the approaches are complementary: both rely heavily on a strong theoretical model with one-way causal flow, and make explicit the distinctions between context, mechanism and outcome variables [17]. Ideally, theory-driven evaluations should have both theoretical and empirical components [4]. A ToC already includes indicators for each step and therefore the ToC and path analysis approaches may be combined, making the theory accountable to empirical testing [17].

In the context of a shared sanitation trial in Mozambique, we describe the results of a cross-sectional process evaluation assessing intervention implementation, participant response and impacts on hypothesised intermediary outcomes on the pathway from intervention to trial outcomes. We then demonstrate how path analysis can be used to test the intervention ToC, with the aim of understanding how any change in intermediary outcomes may have occurred.

### Study setting: shared sanitation in Maputo, Mozambique and the MapSan trial

Sanitation plays an essential role in preventing transmission of faecal pathogens that cause infectious disease and is associated with improvements in various health outcomes [18]. Improved sanitation facilities shared between multiple households are defined by the UNICEF and World Health Organization Joint Monitoring Programme as ‘limited’ sanitation [19]. Prevalence of shared sanitation is highest in Sub-Saharan Africa [19], where rapid growth of peri-urban informal settlements, characterised by extreme poverty, lack of infrastructure, and high burden of disease [20], poses particular challenges for health. Approximately 70% of Maputo’s residents live in informal settlements [21], 89% use onsite, non-sewered sanitation [22] and 9% of Mozambique’s urban population rely on shared sanitation facilities [19]. Ensuring access, use and quality of shared facilities is important to maximise the health benefits of sanitation. However, ensuring adequate access and quality of shared facilities is often difficult to maintain [23–25].

The Maputo Sanitation (MapSan) trial is a controlled before-and-after study assessing the health impacts of a shared sanitation intervention implemented by Water & Sanitation for the Urban Poor (WSUP) in 11 *bairros* (neighbourhoods) in low-income informal urban settlements of Maputo (clinicaltrials.gov NCT02362932 [26]). Housing in these *bairros* is often organised into compounds, groups of houses clustered around a communal space. Compound members may be homeowners or renters, may share a single latrine, and may have a *chefe de composto* (hereafter *chefe*), an informal leader for the compound who may be responsible for managing sanitation facilities.

The MapSan trial intervention consisted of improved, pour-flush toilets with a septic tank shared among all compound members, delivered in two forms to meet a target ratio of one cabin (i.e. toilet stall) per 20 users: single-cabin shared latrines (SLs) were intended for 20 or fewer users; Communal sanitation blocks (CSBs), with multiple cabins, were intended for more than 20 users. WSUP planned to construct 200 SLs and 50 CSBs with available funds. CSBs also had a built-in handwashing sink (not by default connected to a water supply) among other amenities (Text A1, Additional file 1). Compound eligibility criteria to receive the WSUP intervention included location, minimum 12 members, poor condition of existing sanitation, willingness to contribute to costs (10%, around US$97, for CSBs; 15%, around US$64, for SLs) [27] and engineering and construction considerations. Existing pit latrines (of varying structure and low quality) were removed, including both the superstructure and pit. New facilities constructed under WSUP supervision were handed over to users during a 14-month period from 2015 to 2016. WSUP mobilised local community-based organisations (CBOs) to identify possible intervention sites and conduct compound-level training and household-level hygiene promotion. For the MapSan trial, control compounds meeting a subset of intervention criteria and of comparable compound population were enrolled concurrently with intervention compounds across 17 *bairros* of Maputo, including the 11 intervention *bairros*. Children aged between 29 days and 48 months were enrolled at baseline (pre-intervention) [26]. Further details of eligibility and enrolment are available [26, 28, 29].

**Fig. 1:**
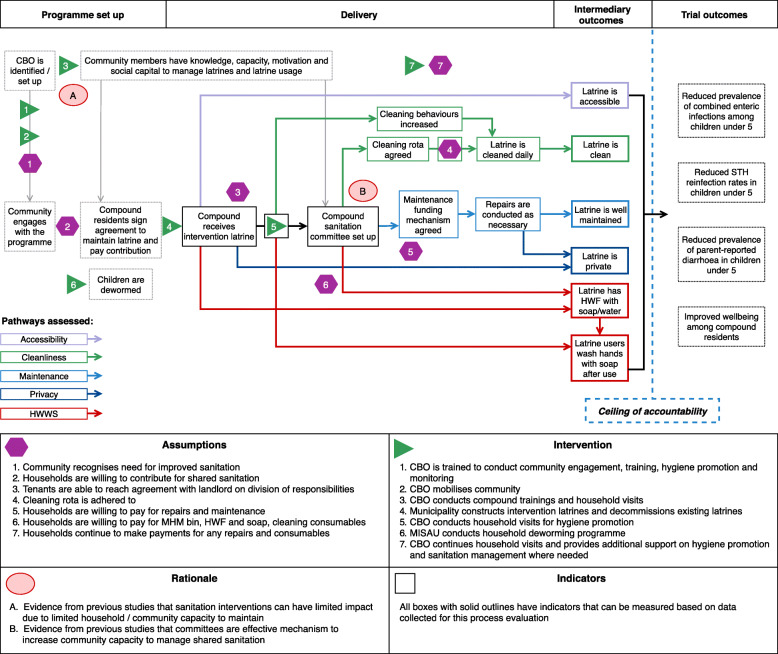
Theory of Change for the MapSan intervention in Maputo, Mozambique. Abbreviations: CBO, community-based organisation; HWF, handwashing facility; HWWS, handwashing with soap; MHM, menstrual hygiene management; MISAU, Ministério da Saúde (Ministry of Health) Mozambique; STH, soil-transmitted helminths

The ToC (Fig. 1) describes how intervention activities were hypothesised to promote changes in behaviour leading to intermediary outcomes, positioned on the causal pathway to trial outcomes. This ToC was *not* formally developed by the intervention agency (WSUP) before the intervention; rather, it was re-constructed *post hoc* based on structured conversations with WSUP programme managers, and then reviewed by these managers to ensure it accurately described the sequence of intervention activities. The rationale behind the theory and underlying assumptions are also presented. In essence, the ToC for MapSan was that high-quality shared sanitation facilities would be installed, and subsequent CBO visits within intervention compounds would lead to formation of compound sanitation committees and financing agreements, creating the necessary capacity to manage sanitation facilities over time. Effective shared sanitation management, maintenance, and promotion of handwashing behaviours through household-level visits were expected to promote child health and user wellbeing in intervention compounds.

A 2016 external evaluation of the WSUP sanitation project [30] assessed relevance, efficiency and sustainability of the intervention through focus-group discussions with beneficiaries, local *bairro* leaders, and CBOs. However, this external evaluation did not quantitatively assess effects on participant response nor compare with control compounds of the MapSan trial. The report noted a major scaling-back of planned hygiene promotion activities due to increased costs of infrastructure construction, suggesting that the effectiveness of this component warrants investigation. A qualitative assessment of sanitation management structures in study compounds [31] identified factors including compound leadership*,* number of households and relational structure that might influence cleanliness and maintenance of facilities.

## Methods

### Evaluation design

This evaluation comprised a cross-sectional study of intervention and control compounds of the MapSan trial between 24- and 42-months post-intervention, and was centred on the trial ToC (Fig. 1). Drawing on relevant process evaluation frameworks [2, 32, 33], we assessed three domains with several subdomains (labelled A–D):

1. Implementation fidelity – *how intervention dose (A) and reach (B) compared with what was intended*
2. Participant response – *whether participant behaviours (C) and intermediary outcomes (D) changed as intended*
3. Context – *whether implementation and response were modified by external factors*

Indicators used to assess each domain are detailed in Additional file 2. Subdomains of dose received, participant behaviours and intermediary outcomes (A, C and D) map directly to indicators on the ToC. Examination of context focused on demographic characteristics of clusters and participants, how those may have affected intervention reach (B), and are incorporated as exogenous variables in subsequent path models. Analytic methods focused on the contribution of fidelity, response, and context to intermediary outcomes, including: sanitation accessibility, privacy, cleanliness and maintenance, presence of a handwashing facility (HWF) with access to soap and water, and handwashing with soap (HWWS) by users.

### Data collection and sampling

Enumerators collected survey data between 26 April and 17 July 2018. Compound eligibility criteria included having households that had completed the 12-month follow-up phase of the MapSan trial. In each selected compound, up to three respondents were recruited (Text A2, Additional file 1), including caregivers of children enrolled in the MapSan trial (‘MapSan trial participant’) an additional adult respondent not enrolled in the MapSan trial (‘secondary respondent’), and compound *chefes* at half the compounds, identified by residents as the person with the most knowledge about the compound’s sanitation management.

Surveys were conducted using mWater*,* developed for this study in English, translated to Portuguese, and piloted over 1 week in December 2017 and an additional 2 days in April 2018 in non-study compounds of one Maputo *bairro*. Feedback on survey items from local enumerators in Maputo was used to iteratively revise the surveys and ensure face validity*.* All data were encrypted and kept on a secure server.

Surveys were developed specifically for this process evaluation and addressed various aspects of intervention implementation and participant behaviours, and demographic characteristics of individuals and compounds (Additional file 3). Photographs of sanitation infrastructure taken by enumerators were checked against responses to ensure they matched reported intervention status.

### Data handling and analysis

Data were cleaned and analysed in Stata version 16.0 (StataCorp, College Station, TX, USA).

Intervention and control compounds were designated as in the MapSan trial. Control compounds that received intervention latrines and intervention compounds that did not receive intervention latrines were analysed under intervention fidelity and excluded from subsequent analyses. Control compounds that had upgraded their sanitation autonomously following initial recruitment to the trial were still included as controls.

We derived measures of dose received, reach, and participant behaviours (subdomains A, B and C) from questionnaire responses from all respondents. The proportion of participants who reported personally cleaning the latrine twice per week was used to indicate individual cleaning frequency, and the proportion reporting their latrine being cleaned daily was used to indicate collective cleaning frequency.

We derived measures of pre-defined intermediary outcomes (listed above and in Additional file 2) from questionnaire responses and observation of facilities linked to household respondent (MapSan trial participant and secondary respondent) surveys. The condition of the latrine slab/floor was used as a proxy measure for latrine maintenance. Latrines were considered ‘clean’ if no faeces, solid waste, urine, dirty water or anal cleansing materials were visible, ‘accessible’ if they had no outside lock or all households had a key, and ‘private’ if they had a working door and inside lock. The presence of soap residue (or signs of recent soap use) at an HWF was used as proxy for HWWS.

For compound-specific indicators (e.g. whether the compound received household visits), we applied a single respondent’s data to all respondents from that compound, prioritizing data from the *chefe*, followed by the MapSan trial participant [34]. For individual-level outcomes (e.g. whether the respondent participated in training), we included all respondents in analysis, and considered respondent type as a covariate, using the intraclass correlation coefficient (ICC (1,k)) to indicate interrater reliability, with values above 0.4 indicating fair agreement [35]. Intermediary outcomes were considered individual-level responses, considering that sanitation quality and HWWS may vary even for the same facilities, depending on the day of visit.

We calculated relative household wealth for household respondents using the Simple Poverty Scorecard for Mozambique [36], including 8 of 10 inputs and excluding number of beds (missing data) and latrine type [37]. Surveys with compound *chefes* were limited to sanitation operation and management issues and did not include poverty scoring measures. Since *chefe* is not a formal leadership role and was often the person resident in the compound for the longest time, we approximated household wealth for *chefes* as the average of other respondents’ scores within the same compound. We converted scores into relative wealth terciles for analysis.

We compared intermediary outcomes and indicators of fidelity and participant behaviours between intervention and control compounds using the chi-squared (χ^2^) test.

### Path analysis

We converted all variables included to dichotomous variables (Additional file 2). As some indices were only available for a subset of the population by planned missingness (i.e. intermediary outcomes assessed for household respondents and not *chefes*), we applied full information maximum likelihood estimation. There were no other missing data.

**Fig. 2:**
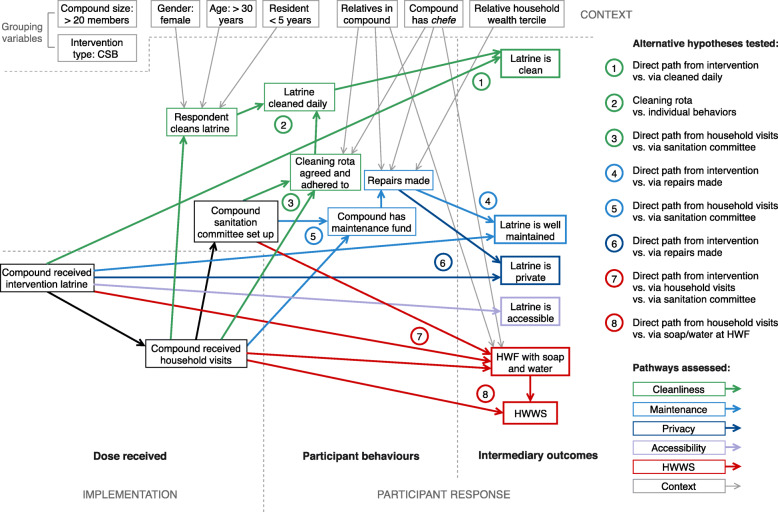
Initial structural model constructed from the MapSan intervention theory of change. This model formed the basis of path analysis of cross-sectional data from MapSan trial compounds. Sections of the model corresponding to process evaluation domains (implementation fidelity, participant response, context) and subdomains (dose received, participant behaviours, intermediary outcomes) indicated by dashed lines. Abbreviations: ‘*chefe’*, *chefe de composto* (informal compound leader); CSB, communal sanitation block; HWF, handwashing facility; HWWS, handwashing with soap

We converted the ToC (Fig. 1) into an initial path diagram (Fig. 2) designed to test ToC, including the role of specific elements (e.g. sanitation committee, cleaning rota), and the overall relative effects of sanitation infrastructure (direct effects) and hygiene promotion activities (indirect effects) on intermediary outcomes. We initially included all paths in the model, and conducted a model trimming process: where multiple explanatory variables led to the same endogenous variable (e.g. intervention facilities and collective cleaning both lead to latrine cleanliness), we removed a path where its removal did not alter path coefficients for other explanatory variables and was therefore considered an unnecessary complication. We began at purely endogenous variables (intermediary outcomes) and moved backwards through the diagram (in numbered order in Fig. 2). Longer pathways were therefore removed when they no longer connected to an intermediary outcome. We calculated the variance accounted for by the path model for each endogenous variable using Wright’s tracing rules for path analysis [16], and variables contributing additional error were considered for removal, in order to achieve the simplest abstraction useful for our aims while maintaining the theoretical basis for the model.

We defined a priori a set of contextual factors hypothesized to influence the larger ToC. For each contextual factor, we estimated path coefficients and repeated the above trimming process for each dichotomous group separately, assessing whether the grouping led to changes in model structure or changes to specific path coefficients within the model. Where there were no structural changes, we treated the variable as another exogenous variable within the model at pre-specified points (Fig. 2), assessed alongside other explanatory variables in the larger model trimming process.

By using a carefully-specified ToC with the minimum number of variables kept close to those concepts they represented, we minimised redundant measures and avoided sources of collinearity in our models. We examined models throughout the process for any instability in path coefficients that would indicate the need to merge too-similar conceptual variables.

We conducted path analysis as both a probit model using generalised structural equation modelling (GSEM), and as a linear probability model using SEM, assuming many dichotomous outcomes (e.g. latrine cleanliness) indicate an underlying normal distribution. Both models produced an identical structure and parameter estimates for the linear probability model fell within acceptable ranges (between 0 and 1). We therefore retained the linear probability model.

The final model was estimated using clustered robust standard errors, adjusting for non-independence within compounds. Use of robust standard errors precluded use of likelihood-based indices of goodness-of-fit, so we report the coefficient of determination for the final model. The model was refined using standardised coefficients and reported with unstandardised coefficients for interpretability. We calculated indirect effects as the product of unstandardised path coefficients along each pathway from intervention to intermediary outcomes, and total effects as the sum of indirect and direct effects.

## Results

**Fig. 3:**
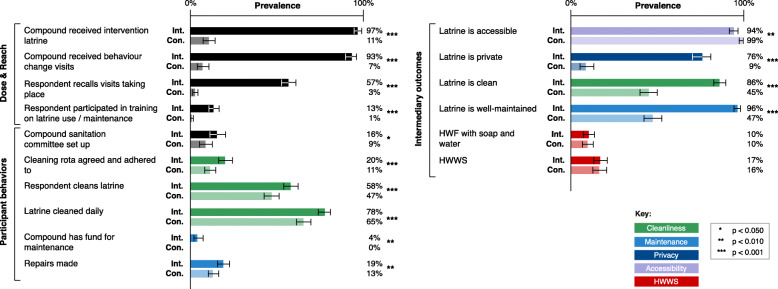
Prevalence of implementation fidelity and participant response indicators between intervention and control compounds of the MapSan trial. Associated 95% confidence intervals and significance level of the chi-squared test comparing prevalence between trial arms indicated. Abbreviations: Con, control; HWF, handwashing facility; HWWS, handwashing with soap; Int, intervention

Evaluation findings for context (demographics), implementation fidelity and participant response are presented. Implementation and response indicators were cross-tabulated with the trial arm and tested for associations using the χ^2^ test (Fig. 3).

### Respondents and context

We sampled 854 household respondents (389 MapSan trial participants, 465 secondary respondents) and 300 compound *chefes*, of whom 842 (99%) and 295 (98%) consented to an interview, respectively, from 556 compounds (279 intervention, 277 control). Data from all respondents was used to assess fidelity of implementation (further details provided below). After assessing fidelity of implementation, we excluded intervention compounds that *had not* received the intervention and control compounds that *had* received the intervention. After exclusions, there were 1049 responses from 517 compounds (270 intervention, 247 control) (Table 1).

**Table 1:** Characteristics of respondents and their residential compounds from a cross-sectional survey in MapSan trial compounds

**Respondent characteristics**	**Total**	**MapSan trial arm**		**Respondent type**	
		**Intervention**	**Control**	**Household respondent**	***Chefe de composto***
**N**	1049	542	507	777	272
**Females,** n (%)	767 (73)	392 (72)	375 (74)	621 (80)	146 (54)
**Age**, median years (IQR)	34 (26, 48)	36 (27, 50)	32 (25, 43)	32 (25, 43)	43 (32, 55)
**Length of residence**, median years (IQR)	18 (5, 30)	20 (8, 32)	12 (5, 27)	12 (5, 26)	26 (14, 38)
**Rents home,** n (%)	382 (36)	175 (32)	207 (41)	314 (40)	68 (25)
**Relatives in compound**^a^, n (%)	148 (19)	80 (20)	68 (18)		
**Wealth tercile**^a^, n (%)					
*1st*	349 (34)	173 (33)	176 (35)		
*2nd*	334 (33)	187 (35)	147 (30)		
*3rd*	341 (33)	168 (32)	173 (35)		
		**MapSan trial arm**			
**Compound characteristics**	**Total**	**Intervention**	**Control**		
**N**	517	270	247		
**Compound members,** median (IQR)	13 (9, 18)	13 (9, 18.5)	13 (9, 18)		
**Members < 5 years old**, median (IQR)	2 (1, 3)	2 (1, 3)	2 (1, 3)		
**Compound has a** ***chefe***^a^, n (%)	270 (55)	144 (56)	126 (53)		

^a^Based on responses from household respondents only

Household respondents were predominantly women (80%) with median age 32 years; most *chefes* sampled were women (54%). Approximately one-third (36%) of respondents rented, with landlords not resident in the same compound for most of these (63%). Residents of larger compounds (> 20 members) were less likely to have family connections within the compound (8% vs. 21%; Pearson’s χ^2^(1 df) =14, *p* < 0.001).

### Implementation fidelity

#### Dose received

Overall, the sanitation infrastructure was implemented as intended (Fig. 3): 97% of intervention compounds (270/279) received intervention latrines, of which 219 (81%) were SLs and 51 (19%) were CSBs, and 92% were installed by the end of 2016, in line with implementation timelines. Although 23% of intervention compounds where a respondent was present at delivery reported user capital contributions causing delay to installation, there was no association between reported delays and year of implementation (χ^2^(5) = 4.7, *p* = 0.45). Thirty control compounds (11%) had received intervention latrines (excluded). After exclusions, eight intervention compounds (3%) also had a non-intervention latrine in use by members.

**Fig. 4:**
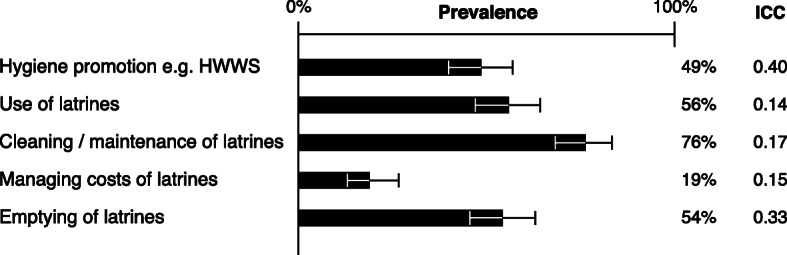
Reported topics discussed at household-level behaviour change visits to intervention compounds of the MapSan trial. Prevalence, 95% confidence interval, and the intraclass correlation coefficient (ICC (1,k)) associated with each response are displayed. *Abbreviations: HWWS, handwashing with soap; ICC, intraclass correlation coefficient*

Similarly, 93% of intervention compounds (252/270) received the household-level behaviour change intervention component, involving a median of three visits (range 1–8), most occurring between 2016 and 2018 – outside the timeframe of the external evaluation [30]. Respondents reported that trainings covered various topics (Fig. 4). Cleaning/maintenance of latrines was the topic most widely discussed at visits (76% of compounds). Some control compounds (17; 7%) received hygiene promotion visits.

#### Reach

Not all of the original criteria for construction of intervention latrines (including location, minimum 12 members, poor sanitation conditions, contribution to costs) were still met by compounds that had received intervention latrines at the time of our survey: only 56% (150/270) had at least 12 members, and 57% (29/51) of CSBs (intended for compounds with over 20 users) had over 20 users, higher than for SLs (6%; 13/219). However, compounds that received household-level behaviour change visits were broadly similar to those that did not (Text A3, Additional file 1).

Among compounds receiving behaviour change visits, there was fair agreement among compound members as to whether visits took place (ICC = 0.45), but not all participated in training (ICC = 0.28). Recall of topics discussed varied among compound members, with ICC values as low as 0.14 for discussion of latrine use (Fig. 4). Overall, 57% (292/542) of intervention respondents recalled visits and 13% (73/542) participated, and recall of visits and participation in training differed across several demographic characteristics (Text A3, Additional file 1).

### Participant response

#### Intermediary outcomes

The intervention achieved improvements to most intended intermediary outcomes (Fig. 3). Compared to control latrines, intervention latrines were much more likely to be private i.e. have a working door and inside lock (76% vs. 8% control; χ^2^(1) = 500, *p* < 0.001), almost twice as likely to be observably clean (86% vs. 45%; χ^2^(1) = 150, *p* < 0.001), and twice as likely to be well-maintained i.e. have a slab/floor in good condition (96% vs. 47%; χ^2^(1) = 240, *p* < 0.001). However, only 10% (78/777) of latrines had an HWF with both soap and water, regardless of trial arm, and few intervention latrines had signs of soap use at an HWF (17%, 67/399), with no difference from control (χ^2^(1) = 0.12, *p* = 0.729). While intervention latrines were more likely to have an outside lock (26% vs. 5%; χ^2^(1) = 66, *p* < 0.001), not all households had a key, resulting in fewer intervention respondents having access to their latrine than control respondents (94% vs. 99%; χ^2^(1) = 12, *p* = 0.001). Shared latrines were not public: only 1% (6/777) of latrines were regularly used by people outside the compound, roughly equal across trial arms.

#### Participant behaviours

Although improvements to intermediary outcomes were expected to result from formalised actions at the compound level, very few compounds formed a committee to manage sanitation, with slightly more in intervention compounds (16% vs. 9% control; χ^2^(1) = 6.0, *p* = 0.014). Compounds were unlikely to have and adhere to a formal rota for cleaning latrines (11% intervention vs. 5% control; χ^2^(1) = 6.1, *p* = 0.013), or to form a fund for sanitation maintenance and repairs – occurring in 4% (11/ 270) of intervention compounds and no control compounds.

Nonetheless, individual cleaning frequency (surveyed individual personally cleaned the latrine twice/week) was significantly higher among intervention respondents (58% vs. 47%; χ^2^(1) = 14, *p* < 0.001), however data suggest cleaning duties were not shared equally among respondents (ICC = 0.18). Frequent collective cleaning (latrine being cleaned on a daily basis) was also reported more often by intervention respondents (78% vs. 65%; χ^2^(1) = 19, *p* < 0.001). Slightly more intervention respondents reported that money was spent on latrine repairs in the past year (19% vs. 13% control; χ^2^(1) = 6.9, *p* = 0.008).

#### Variation by intervention type

Participant response indicators differed by type of intervention latrine received. Compounds with a CSB were more likely to have a sanitation committee (27% vs. 13% SL; χ^2^(1) = 6.8, *p* = 0.009) and maintenance fund (12% vs. 3%; χ^2^(1) = 9.5, *p* = 0.002), to have and adhere to a cleaning rota (20% vs. 9%; χ^2^(1) = 5.2, *p* = 0.023), and to spend money on repairs (33% vs. 15%; χ^2^(1) = 19, *p* < 0.001). CSBs and SLs were similarly well-maintained, but CSBs were more often private (88% vs. 73% SL; χ^2^(1) = 11, *p* = 0.001) whereas SLs were more often clean (89% vs. 78% CSB; χ^2^(1) = 7.8, *p* = 0.005). More SL users reported individual cleaning (63% vs. 39% CSB users; χ^2^(1) = 22, *p* < 0.001), and collective cleaning (80% vs. 68%; χ^2^(1) = 8.2, *p* = 0.004). SL users were also more likely to have an HWF with soap and water (12% vs. 4% CSB; χ^2^(1) = 4.2, *p* = 0.042) and signs of soap use (20% vs. 4% CSB; χ^2^(1) = 12, *p* < 0.001), despite an HWF being built into the CSB infrastructure.

### Path analysis

Grouped analyses by dichotomised contextual variables produced a different structural model when grouping by compound size (compounds of 20 members or fewer vs. more than 20 members) or by intervention type (CSBs vs. SLs). When stratifying by compound size, there was no further structural change when further grouping by intervention type, so we considered compound size to account for differences between CSBs and SLs. Other contextual variables such as having a *chefe* or relatives in the same compound were included as exogenous variables.

**Fig. 5:**
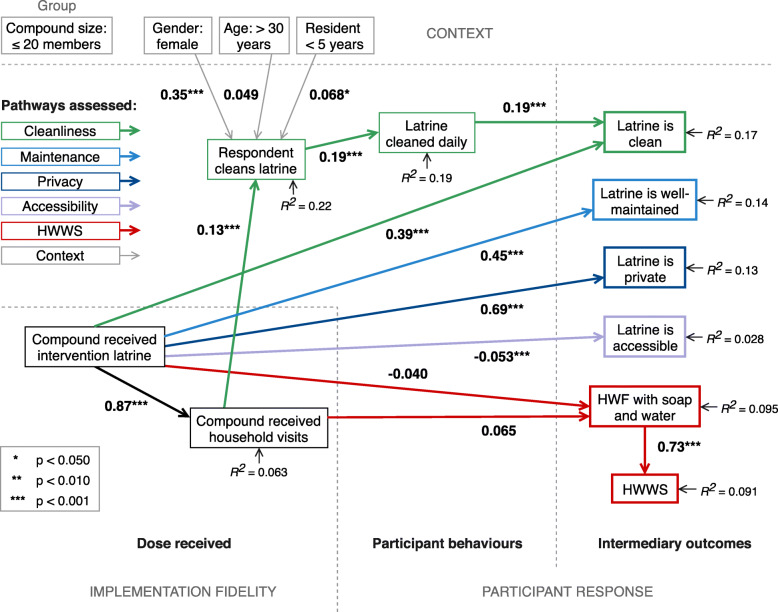
Linear probability path analysis of data from small compounds (≤ 20 members) of the MapSan trial. Path model for small compounds (≤ 20 members) subgroup assessing effects of a sanitation intervention on intermediary outcomes. Unstandardised path coefficients (bold type) represent the increase in absolute probability of the outcome as a result of a unit change in the exposure. Sections of the model corresponding to process evaluation domains and subdomains indicated by dashed lines. The model structure was less complex than that of the large compounds subgroup. Large direct effects on latrine maintenance (condition of slab/floor), privacy (working lock) and cleanliness were observed. Indirect effects on latrine cleanliness via cleaning behaviours were minimal. Intervention latrines were less likely to be accessible to all members of the compound. Abbreviations: HWF, handwashing facility; HWWS, handwashing with soap

**Fig. 6:**
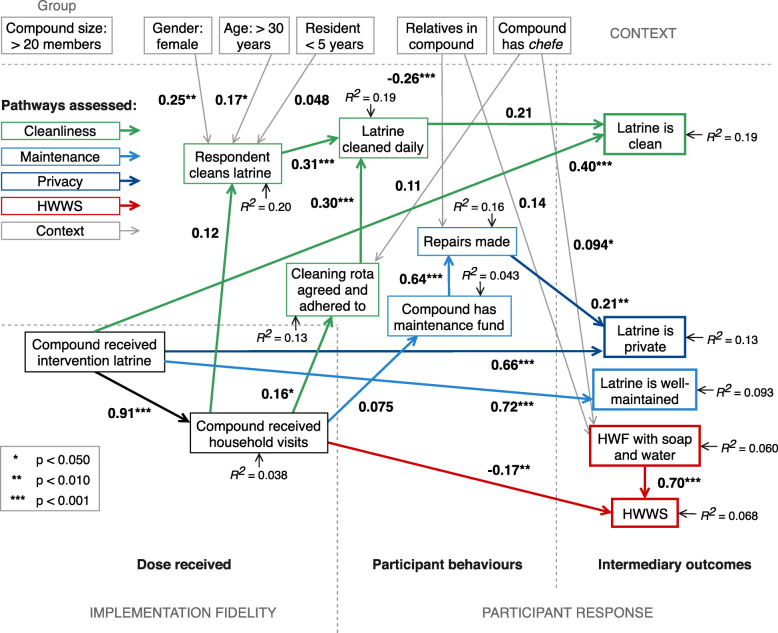
Linear probability path analysis of data from large compounds (> 20 members) of the MapSan trial. Path model for large compounds (> 20 members) subgroup assessing effects of a sanitation intervention on intermediary outcomes. Unstandardised path coefficients (bold type) represent the increase in absolute probability of the outcome as a result of a unit change in the exposure. Sections of the model corresponding to process evaluation domains and subdomains indicated by dashed lines. The model structure was more complex than that of the small compounds subgroup. Large direct effects on latrine maintenance (condition of slab/floor), privacy (working lock) and cleanliness were observed. Indirect effects on latrine cleanliness via cleaning behaviours (supported by a compound cleaning rota system) and indirect effects on latrine privacy were both minimal. A small negative effect on handwashing (soap residue) was observed. Abbreviations: ‘*chefe’*, *chefe de composto* (informal compound leader); HWF, handwashing facility; HWWS, handwashing with soap

Intermediate models (without clustered robust standard errors) for both compound size groups had acceptable fit: for *small compounds* (≤ 20 members), root mean square error of approximation (RMSEA) = 0.066 (90%CI: 0.049–0.083), comparative fit index (CFI) = 0.91, Tucker-Lewis index (TLI) = 0.89); for *large compounds* (> 20 members), RMSEA = 0.063 (90% CI: 0.055–0.070), CFI = 0.93, TLI = 0.91). The final path analysis models for the two groups are displayed in Figs. 5 and 6. The coefficient of determination was 0.839 for small compounds, and 0.897 for large compounds. Unstandardised path coefficients (*b*) represent the amount of expected change – in this case, the increase in absolute probability – in the outcome as a result of a unit change in the exposure. This is considered the *direct effect* of that exposure when controlling for other explanatory variables. The residual variance not explained by the model for endogenous variables is indicated. Covariances between exogenous variables were included in models, but not pictured (full models in Additional files 4, 5, 6 and 7). Estimated total effects (TE), subdivided into direct and indirect effects (IE) where there were multiple pathways, are provided in Table 2.

**Table 2:** Estimated total effects of the MapSan intervention on intermediary outcomes from grouped path analyses

**Compounds with 20 members or fewer**	**Unstandardised coefficient**	**95% CI**	***p*-value**
➔ Accessibility (*direct*)	-0.053	(-0.080, -0.026)	<0.001
➔ Collective cleaning (*indirect*)	0.022	(0.007, 0.036)	0.003
➔ Latrine cleanliness	0.40	(0.32, 0.47)	<0.001
*Direct*	0.39	(0.32, 0.47)	<0.001
*Indirect: via collective cleaning*	0.004	(0.001, 0.007)	0.020
➔ Privacy (*direct*)	0.69	(0.62, 0.75)	<0.001
➔ Latrine maintenance (*direct*)	0.45	(0.38, 0.52)	<0.001
➔ Soap/water at HWF (*indirect*)	0.016	(-0.034, 0.066)	0.519
➔ HWWS (*indirect*)	0.012	(-0.025, 0.049)	0.520
**Compounds with more than 20 members**	**Unstandardised coefficient**	**95% CI**	***p*-value**
➔ Collective cleaning	0.077	(0.009, 0.15)	0.027
*Indirect: via cleaning rota*	0.044	(0.001, 0.087)	0.046
*Indirect: via individual cleaning*	0.033	(-0.013, 0.079)	0.157
➔ Latrine cleanliness	0.41	(0.20, 0.62)	<0.001
*Direct*	0.40	(0.18, 0.61)	<0.001
*Indirect: via collective cleaning*	0.016	(-0.003, 0.035)	0.105
➔ Repairs (*indirect*)	0.044	(-0.007, 0.095)	0.090
➔ Privacy	0.67	(0.50, 0.84)	<0.001
*Direct*	0.66	(0.48, 0.84)	<0.001
*Indirect: via repairs*	0.009	(-0.004, 0.022)	0.161
➔ Latrine maintenance (*direct*)	0.72	(0.55, 0.88)	<0.001
➔ HWWS (*indirect*)	-0.16	(-0.26, -0.048)	0.004

Total effects subdivided into direct and/or indirect effects. Indirect effects are calculated as the product of all coefficients in a pathway. For example, the indirect effect on latrine cleanliness in small compounds is calculated 0.87 * 0.13 * 0.19 * 0.19 = 0.004

*Abbreviations*: *CI* confidence interval, *HWF* handwashing facility, *HWWS* handwashing with soap

Decomposition of covariance between observed variables into ‘direct’ and ‘indirect’ effects suggested that the direct components accounted for the majority of intervention effects on intermediary outcomes due to several factors:

In small compounds, removal of indirect pathways that did not explain variation in outcomes meant large improvements to latrine maintenance (Fig. 5; *b* = 0.45, *p* < 0.001) and privacy (*b* = 0.69, *p* < 0.001) were direct effects. The intervention had a small negative effect on accessibility (*b* = − 0.053, *p* < 0.001), and no significant effects on probability of having an HWF with soap and water or signs of soap use. Where indirect pathways are present, group-specific path coefficients indicate points where the theory breaks down. For example, both the intervention facilities (Fig. 5; *b* = 0.39, *p* < 0.001) and collective cleaning frequency (*b* = 0.19, *p* < 0.001) were associated with latrine cleanliness. However, because the intervention had only a minor effect on collective cleaning (Table 2; TE = 0.022, *p* = 0.003), without use of a rota system, the indirect effect on latrine cleanliness was negligible (IE = 0.004, *p* = 0.020).

In large compounds, the reverse is true for latrine cleanliness. With a greater number of latrine users, collective cleaning frequency does not significantly contribute to latrine cleanliness (Fig. 6; *b* = 0.21, *p* = 0.056), so despite an increase in collective cleaning frequency (Table 2; TE = 0.077, *p* = 0.027), mostly attributable to the compound maintaining a rota system to manage cleaning (TE = 0.044, *p* = 0.046), the effect on cleanliness was also primarily a direct effect of receiving an intervention latrine (*b* = 0.40, *p* < 0.001). Similarly, the intervention had a minimal indirect effect on latrine privacy via recent repairs (IE = 0.009, *p* = 0.161), despite the association between repairs and privacy (*b* = 0.21, *p* = 0.009). Counterintuitively, a negative effect of the intervention on probability of having an HWF with signs of soap use was observed (TE = -0.16, *p* = 0.004), associated with household-level behaviour change activities.

Individual demographic factors influenced individual cleaning in both groups. In small compounds, female respondents (Fig. 5; *b* = 0.35, *p* < 0.001) and those resident in the compound for under 5 years (*b* = 0.068, *p* = 0.049) were significantly more likely to clean the latrine; in large compounds, female respondents (Fig. 6; *b* = 0.35, *p* = 0.001) and respondents above 30 years of age (*b* = 0.17, *p* = 0.027) were more likely to clean. However, compound factors played a significant role only in larger compounds. Presence of a relatives in the compound was negatively associated with repairs (*b* = − 0.26, *p* < 0.001), and presence of a *chefe* promoted availability of soap and water at the HWF (*b* = 0.094, *p* = 0.021).

## Discussion

Process evaluation of a shared sanitation intervention in Maputo revealed that the programme was implemented as intended in terms of dose received and reach, with few controls receiving the intervention and an apparent strong ‘participant response’ in intervention compounds. While the Maputo Sanitation intervention targeted compounds with at least 12 residents, 44% of intervention compounds in this study had fewer residents, due in larger part to significant out-migration in the study area in the period since compound selection (2015) [28]. Among intervention compounds, improvements were made to intermediary outcomes of latrine cleanliness, maintenance and privacy – latrine ‘quality’ – but not to handwashing behaviour as indicated by soap residue. While concerns around quality of shared sanitation have prevented acceptance of shared facilities as ‘improved’ in global indicator classifications [19], this study demonstrates that shared sanitation can be high-quality, as the MapSan intervention was a significant improvement over existing shared latrines. This finding is particularly relevant to residents of low-income urban settlements, where shared sanitation is the only solution in the short-to-medium term [38]. Improvements to intermediary outcomes were not accompanied, however, by compliance with the intervening behaviours hypothesised to mediate change, such as formation of a compound sanitation committee, and variation by intervention type (SL or CSB) was observed. Path analysis applied to ToC gave insights into why change may not have occurred as expected, adding to our understanding of ‘what works, for whom and in what circumstances*’* [6] in this setting.

### ‘What works?’

In our analysis, partitioning the different effects of the intervention suggested that large improvements to sanitation quality (latrine cleanliness, privacy, maintenance) were mainly driven by direct effects of intervention facilities, and not by indirect pathways including participant behaviours. This finding contradicts the ToC, in which intermediary outcomes were considered part of ‘participant response.’ Path coefficients also point to weakness in the ToC. For instance, contributions of compound members towards repairs did not translate into maintenance of latrines, assessed by condition of the slab/floor. This may be due to survey timing – the expected lifetime of intervention latrines is longer than traditional latrines, so the need for repairing the floor may have not arisen [30] – or might reflect limited economic resources reducing capacity to make major repairs [39]. In large compounds, spending towards repairs was associated with maintenance of the latrine door and lock – moving parts expected to wear down – suggesting minor repairs were being made, prioritising privacy. Formation of a compound sanitation committee also did not explain variance in participant behaviours. The committee model may be less appropriate in this setting for compounds with 15 or fewer members, as informal management systems may be sufficient to maintain quality among small groups of people with existing social ties [31].

Several behavioural interventions have demonstrated effectiveness at improving and maintaining the quality of shared sanitation [40–42]. In the longer term, stronger behaviour-focused strategies that prevent reversal of progress on intermediary outcomes associated with infrastructure improvements may be needed [24]. The ineffectiveness of the household-level behaviour change component at altering handwashing behaviour, with an apparent negative effect for some participants, may play a critical role in determining the intervention’s health benefits. Inclusion of outcome variables in the path model could provide a bridge between evaluation of process and mechanisms and outcome evaluation, and inform assessment of intervention health impacts.

### ‘For whom?’

An important distinction emerged between contextual factors that influenced values of specific parameters within the model, and those for which the dynamics of the model were fundamentally altered. Analysis of subgroups suggested structural differences by compound size accounted for disparities by type of intervention received. The large compound model (> 20 members) was more closely aligned to theorised mechanisms, with elements such as having a cleaning rota or maintenance fund playing an important role, but cleaning practices were less effective at maintaining cleanliness. With a high number of users, shared management responsibility is difficult to achieve [43] and facilities can quickly deteriorate; higher numbers of households sharing latrines have been correlated with lower sanitation quality [44]. Identifying factors affecting the dynamics of change has useful application in multi-site evaluations [45], and supports understanding of how interventions function for specific groups and in varied contexts.

### ‘In what circumstances?’

Characteristics of both compounds and individuals were also included in the models as potential modifying factors, allowing testing of hypotheses that emerged from qualitative research. Women were 25–35% more likely to clean latrines, confirming accounts of division of cleaning responsibilities along gendered lines in this and other urban informal settlements [23, 25, 31]. The idea that more transient residents contributed little to cleaning [31] was refuted: length of residence was negatively associated with self-reported individual cleaning frequency. In large compounds, members without family relations in their compound were more likely to spend money on repairs – perhaps unsurprising given repairs were made to maintain latrine privacy – and presence of a compound *chefe* affected soap and water availability and adherence to a cleaning rota, supporting qualitative findings that compound leaders can facilitate collective decision-making and resolve conflict [23, 31]. An alternative approach to improve hygiene behaviours in this setting might therefore focus on training and funding a dedicated compound resident to promote change.

### Limitations

This study was cross-sectional and examined only a portion of the ToC 24–42 months after intervention delivery; therefore, the causal direction could not be determined, nor variation in intervention effects or demographics over time. Building in data collected at multiple time points – for example, measuring change in behaviour across the intervention period – could improve validity [46] and allow assessment of longevity of effects. Observation of handwashing may be a better indicator of behaviour than presence of soap residue, as soap may be used for other purposes than handwashing. We considered contextual factors only at a limited level, and participants were similar with regard to demographics like socioeconomic status, so we could not adequately assess their influence. Our study focused primarily on participants in a larger trial of a public health intervention and findings may not be generalisable to non-trial participants, as will often be the case with SEM [47].

Some limitations may be minimised by developing a ToC before the intervention is implemented, specifying a set of indicators appropriate for the expected agents of change and the level at which the intervention was implemented, and clearly defining key contextual factors. Whereas path analysis assumes variables are measured without error, including latent variables in SEM may be appropriate to assess underlying changes to participants’ knowledge, attitudes or social norms, and could allow measurement error to be estimated and controlled [48]. Use of a linear probability model as in this example will not often be appropriate, and evaluators should make use of GSEM options depending on the variables of interest.

Lastly, while path analysis applied to intervention ToC can provide useful evidence to validate or negate proposed theory, there are many assumptions involved with path analysis or SEM – that pathways are independent [49], all relevant variables are included and the model is well-specified – that limit its widespread use and adoption. The assumptions underlying SEM may not always be met and findings from this and similar analyses should be interpreted with caution. It is plausible that direct effects of shared sanitation might be further decomposed into more likely pathways, such as the direct effect of infrastructure – the ‘behaviour setting’ [50] – on cleaning behaviour. Uncertainty around paths to handwashing behaviours means the negative effect for some participants remains unexplained. Path analysis is not designed for exploratory purposes of discovering other factors that may be relevant to the system. Adding or removing variables *a posteriori* to maximise model fit can also lead to over-specification or path coefficients that are heavily dependent on the correlation structure of predictors [51]. Ultimately, the capacities of path analysis to test a Theory of Change are dependent on the validity of the theories being evaluated. Close coordination with qualitative research activities [17], as recommended in process evaluation guidance [2], is therefore necessary to include the most plausible set of alternative pathways. Qualitative evidence could also be used to develop theory concerning unintended consequences [52], which could be fed back into the path model for testing. Properly placed in regard to other sources of evidence, combining path analysis with ToC can be an effective tool for unpacking causal pathways for complex interventions.

## Conclusions

Although identification of process indicators and specification of a causal model are inherent parts of the process of developing a ToC, path analysis and related methods have been under-utilised in testing the theorised causal model. Application of the path analysis approach to a shared sanitation intervention in Mozambique revealed weaknesses in the proposed ToC and provided evidence for different mechanisms of change between contextual subgroups, informing interpretation of process evaluation findings. The MapSan intervention achieved high implementation fidelity and improved intermediary outcomes related to sanitation quality, likely through direct effects of sanitation infrastructure and not behaviour change activities. For process evaluations of complex interventions with multiple interacting components, this approach has use in distinguishing essential components, supporting refinement of intervention theory and improving understanding of the contexts in which an intervention will be most effective.

## Supplementary Information

**Additional file 1: Text A1.** Details of MapSan intervention facilities. **Text A2.** Sampling procedures. **Text A3.** Further details on intervention reach.

**Additional file 2.** Process evaluation domains, subdomains and indicators. Process indicators used, with details of dichotomisation and role of variables in path models (exogenous / endogenous, intermediary outcome / potential mediator).

**Additional file 3. **Questionnaire developed for the MapSan process evaluation*.* Portuguese with English translation.

**Additional file 4. **Generalised structural equation model with probit link using data from small compounds (≤ 20 members)*.* Abbreviations: HWF, handwashing facility; HWWS, handwashing with soap.

**Additional file 5. **Generalised structural equation model with probit link using data from large compounds (> 20 members)*.* Abbreviations: ‘*chefe*’, *chefe de composto* (informal compound leader); HWF, handwashing facility; HWWS, handwashing with soap.

**Additional file 6.** Linear probability path analysis of data from small compounds (≤ 20 members) – standardised coefficients and covariances between exogenous variables. Abbreviations: HWF, handwashing facility; HWWS, handwashing with soap.

**Additional file 7. **Linear probability path analysis of data from large compounds (> 20 members) – standardised coefficients and covariances between exogenous variables. Abbreviations: ‘*chefe*’, *chefe de composto* (informal compound leader); HWF, handwashing facility; HWWS, handwashing with soap.

## Data Availability

The datasets used and analysed during the current study are available from the corresponding author on reasonable request.

## Abbreviations

CBO: Community-based organisation
CFI: Comparative fit index
CSB: Communal sanitation block
GSEM: Generalised structural equation modelling
HWF: Handwashing facility
HWWS: Handwashing with soap
ICC: Intraclass correlation coefficient
IE: Indirect effect
MapSan: Maputo sanitation
MRC: Medical Research Council
NICE: National Institute for Health and Care Excellence
RMSEA: Root mean square error of approximation
SEM: Structural equation modelling
SL: Shared latrine
TE: Total effect
TLI: Tucker-Lewis index
ToC: Theory of change
WSUP: Water & Sanitation for the Urban Poor
