# Supplementary material for: Using path analysis to test theory of change: a quantitative process evaluation of the MapSan trial
Source: BMC Public Health. 2021 Jul 16;21:1411. doi: 10.1186/s12889-021-11364-w (PMC8285873; doi:10.1186/s12889-021-11364-w)
Supplement: Supplementary file 1 — Additional file 1: Text A1. Details of MapSan intervention facilities. Text A2. Sampling procedures. Text A3. Further details on intervention reach. [file 12889_2021_11364_MOESM1_ESM.docx]

## **Additional file 1:** Supplementary text

**Text A1**: Details of MapSan intervention facilities

The MapSan trial intervention consisted of improved, pour-flush toilets with a septic tank. Septic tank design, placement, and soakaway pits were custom-designed by WSUP based on site constraints and number of users. Communal sanitation blocks (CSBs), designed for more than 20 users, also had an external water storage tank and tap, for which users had to independently arrange and pay for piped connection to the municipal water authority, a concrete laundry basin, rainwater harvesting tank, and a handwashing sink that drained to the septic tank. CSBs in compounds with over 60 residents had a urinal on an external wall that drained to the septic tank.

**Text A2:** Sampling procedures

The ‘MapSan trial participant’ was the primary caregiver of a child enrolled in the MapSan trial. Because these participants represented a relatively homogenous group, a ‘secondary respondent’ was identified as another adult residing in the compound for more than 30 days, sampled from the third household on the right of the compound entrance. If no respondent was available, the next third house on the right was selected. In a random selection of half of all sampled compounds, we recruited the compound *chefe*, identified by residents as the person with the most knowledge about the compound’s sanitation management (usually the person who had lived in the compound the longest). The *chefe* survey was intended to provide reliable information on compound-level programmatic elements and did not include facility observations.

**Text A3:** Further details on intervention reach

Compounds that received household-level behaviour change visits were broadly similar to those that did not, with respect to *bairro* (χ^2^(9)=9.8, p=0.363), compound size (>20 members vs. ≤20 members; χ^2^(1)=1.5, p=0.226), relative wealth tercile (χ^2^(2)=3.3, p=0.196) and intervention type (CSB vs. SL; χ^2^(1)=2.2, p=0.135).

Respondents more likely to recall visits were men (63% vs. 53% of women; χ^2^(1)=3.9, p=0.049), and those resident in the compound for over five years (61% vs. 42% of those resident for under five years; χ^2^(1)=16, p<0.001). CSB users were more likely than SL users to recall visits (73% vs. 53%; χ^2^(1)=16, p<0.001) or have participated in training (20% vs. 13%; χ^2^(1)=4.4, p=0.037), and participation varied at the *bairro* level (χ^2^(12)=41, p<0.001).
