# Supplementary material for: Using path analysis to test theory of change: a quantitative process evaluation of the MapSan trial
Source: BMC Public Health. 2021 Jul 16;21:1411. doi: 10.1186/s12889-021-11364-w (PMC8285873; doi:10.1186/s12889-021-11364-w)
Supplement: Supplementary file 2 — Additional file 2. Process evaluation domains, subdomains and indicators. Process indicators used, with details of dichotomisation and role of variables in path models (exogenous / endogenous, intermediary outcome / potential mediator). [file 12889_2021_11364_MOESM2_ESM.docx]

## **Additional file 2:** Process evaluation domains, subdomains and indicators

| Domain & subdomain |  |  |  |  | | | |
| --- | --- | --- | --- | --- | --- | --- | --- |
| Indicator | Study population | Trial arm | Dichotomisation | Role of variable in path model | | | |
| Domain 1: Implementation fidelity | | | | | | |  |
| Subdomain: Dose received (A) | | | | | | |  |
| Number of intervention compounds that received intervention latrines | Single respondent (details in text) | Intervention | (After exclusions) compound received intervention latrine: Yes / No | Exogenous | Exposure |  |  |
| Date of installation of intervention latrines | Single respondent | Intervention |  |  |  |  |  |
| Number of control compounds that received intervention latrines | Single respondent | Control |  |  |  |  |  |
| Number of intervention compounds that received household-level behaviour change visits | Single respondent | Intervention | Compound received household visits:  Yes / No | Endogenous | Potential mediator |  |  |
| Frequency and date of household visits | Single respondent | Intervention |  |  |  |  |  |
| Number of control compounds that received household visits | Single respondent | Control |  |  |  |  |  |
| Frequency of topics discussed at household visits | Single respondent | Int/Con |  |  |  |  |  |
| Subdomain: Reach (B) | | | | | | |  |
| Number of respondents who recall household visits taking place | All respondents | Int/Con |  |  |  |  |  |
| Number of respondents who participated in training | All respondents | Int/Con |  |  |  |  |  |
| Demographics of compounds who received intervention latrines | Single respondent | Intervention |  |  |  |  |  |
| Demographics of compounds who received household visits | Single respondent | Intervention |  |  |  |  |  |
| Demographics of respondents who recall visits | All respondents | Int/Con |  |  |  |  |  |
| Demographics of respondents who participated | All respondents | Int/Con |  |  |  |  |  |
| Frequency of topics recalled | All respondents | Int/Con |  |  |  |  |  |
| Domain 2: Participant response | | | | | | |  |
| Subdomain: Participant behaviours (C) | | | | | | |  |
| Number of compounds with a sanitation committee | Single respondent | Int/Con | Yes / No | Endogenous | Potential mediator |  |  |
| Number of compounds with a fund for latrine maintenance | Single respondent | Int/Con | Yes / No | Endogenous | Potential mediator |  |  |
| Number of compounds that have and adhere to a cleaning rota | Single respondent | Int/Con | Yes / No | Endogenous | Potential mediator |  |  |
| Individual cleaning frequency: how often respondent personally cleans latrine | All respondents | Int/Con | Dichotomised based on median: 2 times per week | Endogenous | Potential mediator |  |  |
| Collective cleaning frequency: how often latrine gets cleaned | All respondents | Int/Con | Dichotomised based on median: daily | Endogenous | Potential mediator |  |  |
| Number of compounds that have spent money on repairs in past year | All respondents | Int/Con | Yes / No | Endogenous | Potential mediator |  |  |
| Subdomain: Intermediary outcomes (D) | | | | | | |  |
| Cleanliness: number of latrines that are visibly clean – no faeces, solid waste, urine, dirty water or anal cleansing materials visible (direct observation) | Household respondents (excluding *chefes*) | Int/Con | Yes / No | Endogenous | Intermediary outcome | |  |
| Maintenance: number of latrines with the slab/floor in good condition (direct observation) | Household respondents | Int/Con | Yes / No | Endogenous | Intermediary outcome | |  |
| Privacy: umber of latrines with a working door and inside lock (direct observation) | Household respondents | Int/Con | Yes / No | Endogenous | Intermediary outcome | |  |
| Accessibility: number of latrines that are not locked from the outside or for which all households have a key | Household respondents | Int/Con | Yes / No | Endogenous | Intermediary outcome | |  |
| Number of handwashing facilities with soap and water available (direct observation) | Household respondents | Int/Con | Yes / No | Endogenous | Intermediary outcome | |  |
| HWWS: number of handwashing facilities with visible soap residue or signs of soap use (direct observation) | Household respondents | Int/Con | Yes / No | Endogenous | Intermediary outcome | |  |
| Number of latrines that are used by non-residents of the compound | Household respondents | Int/Con |  |  |  | |  |
| Domain 3: Context | | | | | | |  |
| Number of compound members | Single respondent | Int/Con | Dichotomised based on CSB criteria: 20 members | Exogenous |  |  |  |
| Number of compounds with a *chefe de composto* (informal leader) | Single respondent | Int/Con | Yes / No | Exogenous |  |  |  |
| Number of compounds with a female *chefe* | Single respondent | Int/Con | Yes / No | Exogenous |  |  |  |
| Respondent’s gender | All respondents | Int/Con | Male / Female | Exogenous |  |  |  |
| Respondent’s age | All respondents | Int/Con | Dichotomised based on approx. median: 30 years | Exogenous |  |  |  |
| Respondent’s length of residence | All respondents | Int/Con | Dichotomised based on 'recent' residence of 5 years | Exogenous |  |  |  |
| Household relative wealth tercile | All respondents | Int/Con |  | Exogenous |  |  |  |

Abbreviations: CSB, communal sanitation block; HWWS, handwashing with soap.
