# Supplementary figures and images for: Using path analysis to test theory of change: a quantitative process evaluation of the MapSan trial
Source: BMC Public Health. 2021 Jul 16;21:1411. doi: 10.1186/s12889-021-11364-w (PMC8285873; doi:10.1186/s12889-021-11364-w)

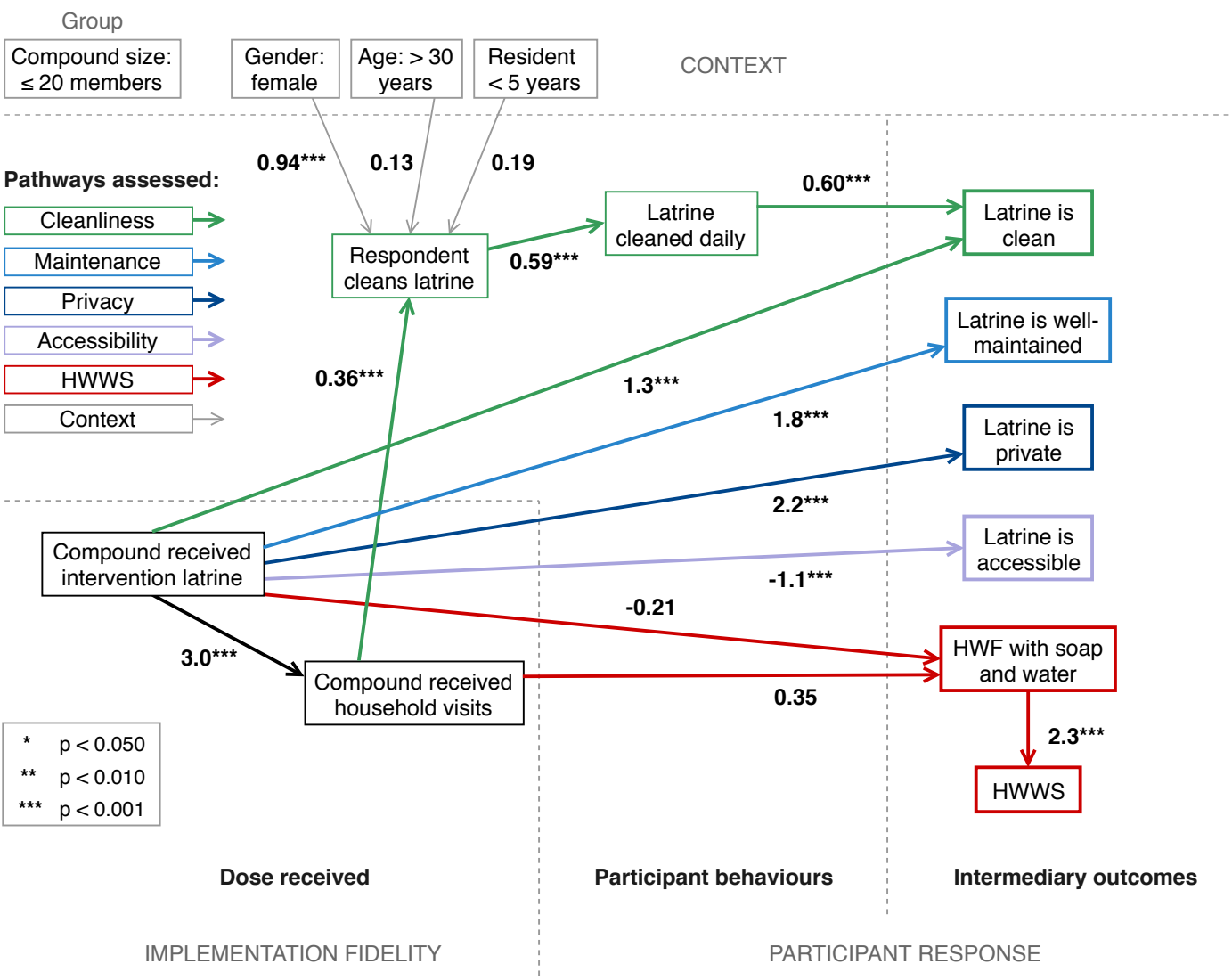

Supplement: Supplementary file 4 — Additional file 4. Generalised structural equation model with probit link using data from small compounds (≤ 20 members). Abbreviations: HWF, handwashing facility; HWWS, handwashing with soap. [file 12889_2021_11364_MOESM4_ESM.pdf]

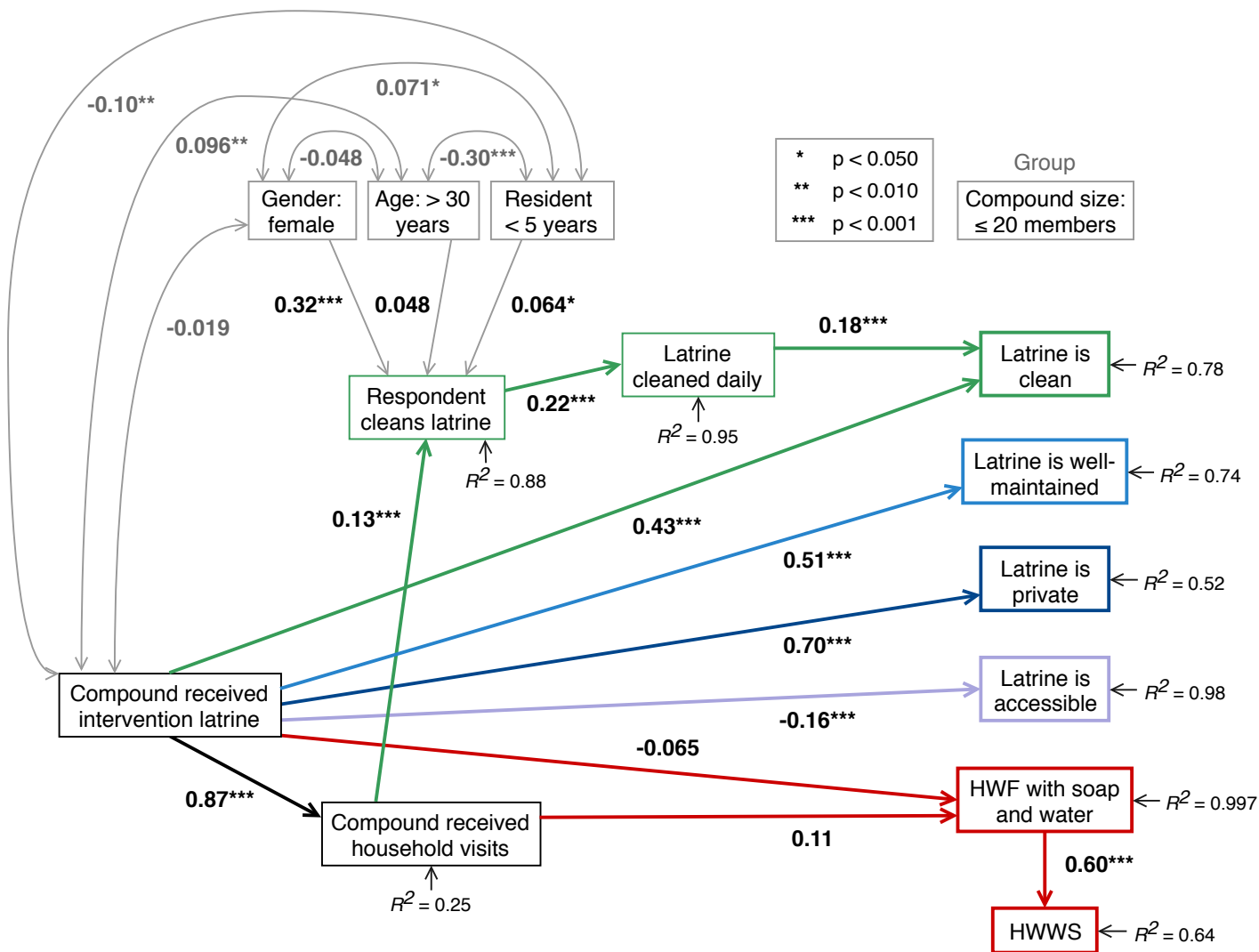

Supplement: Supplementary file 6 — Additional file 6. Linear probability path analysis of data from small compounds (≤ 20 members) – standardised coefficients and covariances between exogenous variables. Abbreviations: HWF, handwashing facility; HWWS, handwashing with soap. [file 12889_2021_11364_MOESM6_ESM.pdf]

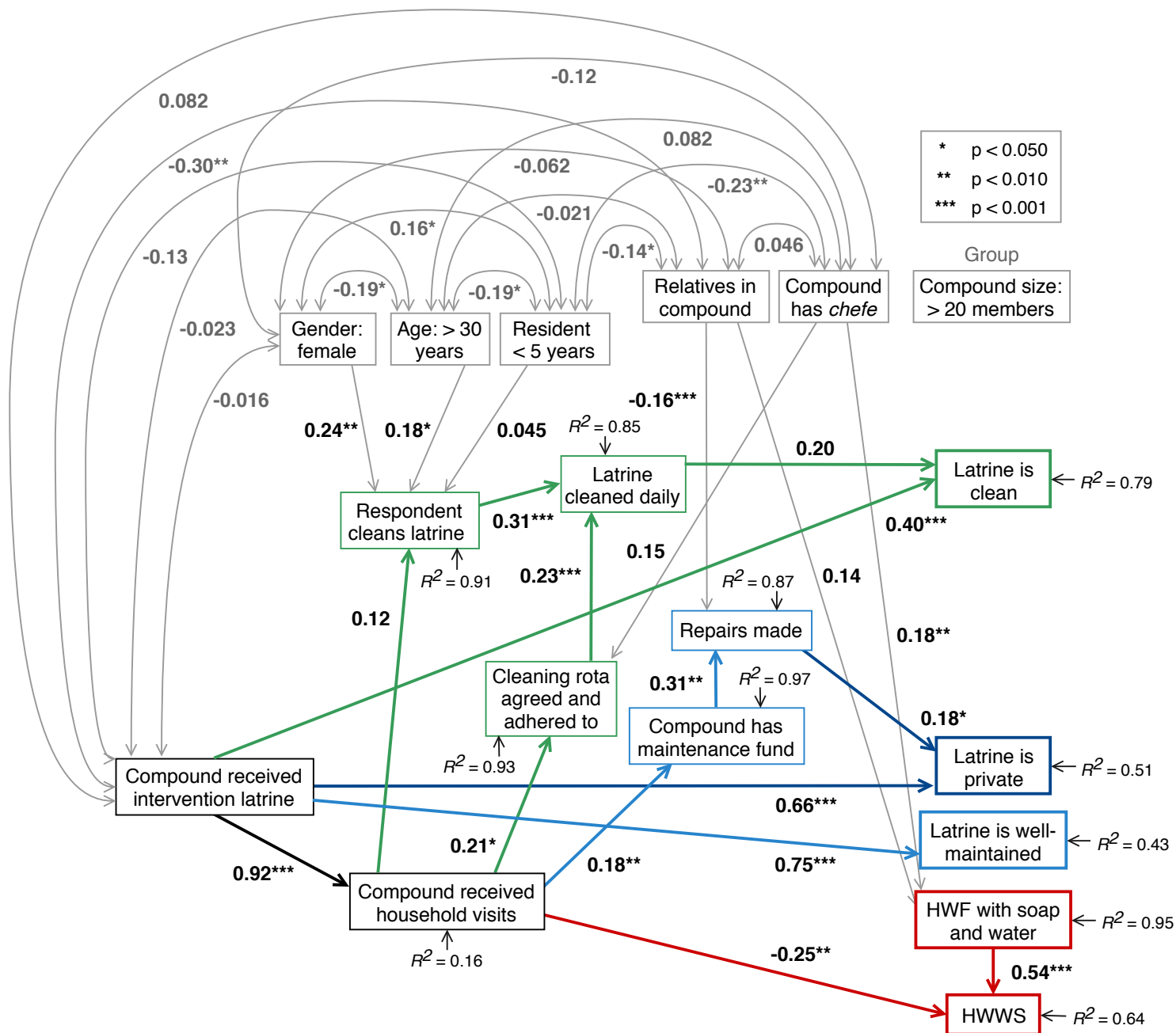

Supplement: Supplementary file 7 — Additional file 7. Linear probability path analysis of data from large compounds (> 20 members) – standardised coefficients and covariances between exogenous variables. Abbreviations: ‘chefe’, chefe de composto (informal compound leader); HWF, handwashing facility; HWWS, handwashing with soap. [file 12889_2021_11364_MOESM7_ESM.pdf]
