# Supplementary material for: Using path analysis to test theory of change: a quantitative process evaluation of the MapSan trial
Source: BMC Public Health. 2021 Jul 16;21:1411. doi: 10.1186/s12889-021-11364-w (PMC8285873; doi:10.1186/s12889-021-11364-w)
Supplement: Supplementary file 5 — Additional file 5. Generalised structural equation model with probit link using data from large compounds (> 20 members). Abbreviations: ‘chefe’, chefe de composto (informal compound leader); HWF, handwashing facility; HWWS, handwashing with soap. [file 12889_2021_11364_MOESM5_ESM.pdf]

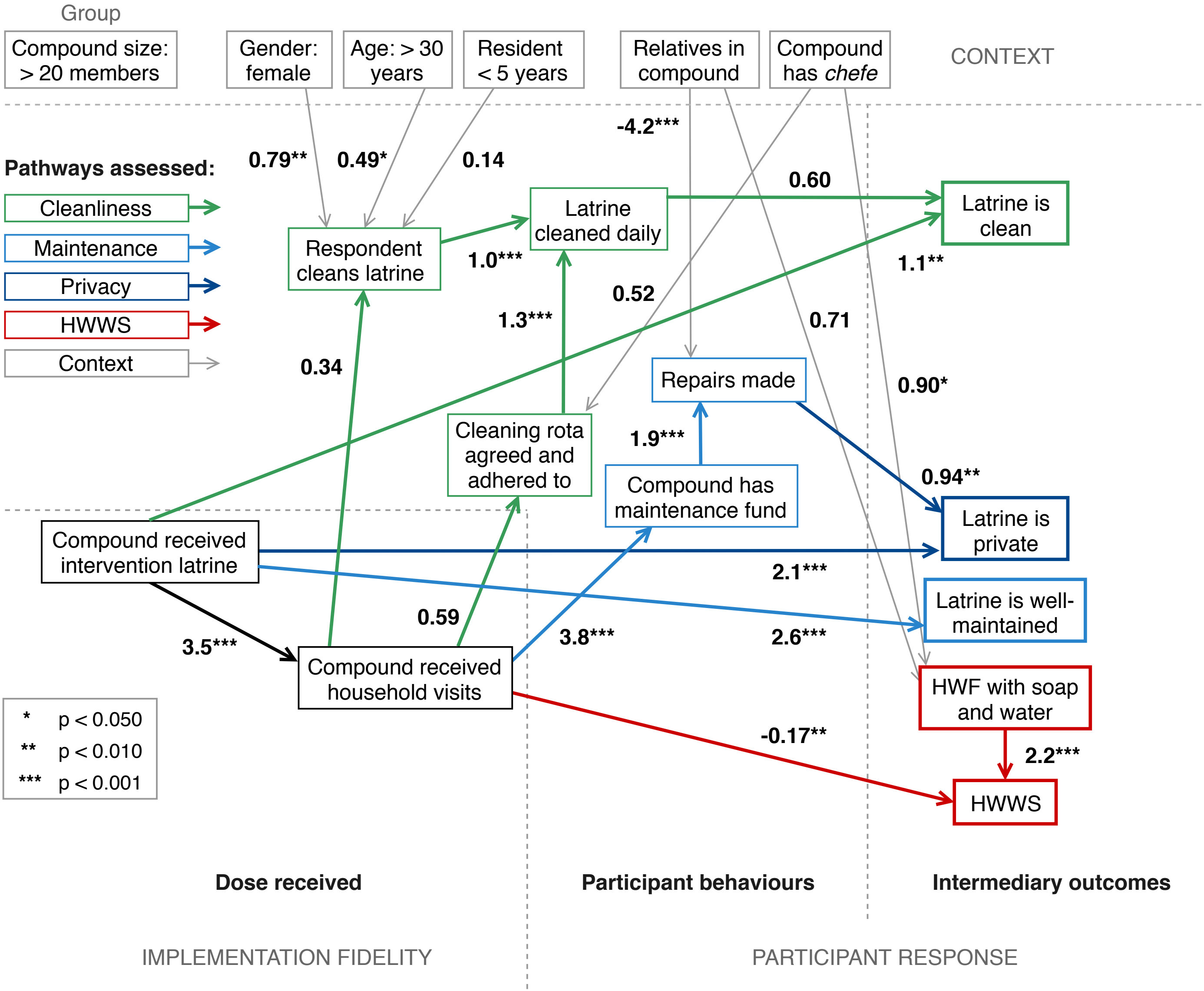

Group
Compound size: > 20 members
Gender: female
Age: > 30 years
Resident < 5 years
Relatives in compound
Compound has *chefe*
CONTEXT
Pathways assessed:
Cleanliness
Maintenance
Privacy
HWWS
Context
0.79**
0.49*
0.14
-4.2***
0.60
Respondent cleans latrine
Latrine cleaned daily
Latrine is clean
1.0***
1.1**
0.52
1.3***
0.71
0.34
Repairs made
0.90*
Cleaning rota agreed and adhered to
1.9***
Compound has maintenance fund
0.94**
Latrine is private
Compound received intervention latrine
2.1***
Latrine is well-maintained
0.59
3.8***
2.6***
3.5***
Compound received household visits
HWF with soap and water
-0.17**
2.2***
HWWS
* p < 0.050
** p < 0.010
*** p < 0.001
Dose received
Participant behaviours
Intermediary outcomes
IMPLEMENTATION FIDELITY
PARTICIPANT RESPONSE
